# Rapid Non-Enzymatic Glycation of the Insulin Receptor under Hyperglycemic Conditions Inhibits Insulin Binding In Vitro: Implications for Insulin Resistance

**DOI:** 10.3390/ijms18122602

**Published:** 2017-12-02

**Authors:** Tyler Rhinesmith, Thomas Turkette, Robert Root-Bernstein

**Affiliations:** Department of Physiology, Michigan State University, 567 Wilson Road, Room 2201, East Lansing, MI 48824, USA; rhinesm1@msu.edu (T.R.); turkette@msu.edu (T.T.)

**Keywords:** diabetic complications, hyperglycemia, insulin receptor, insulin resistance, glycosylation

## Abstract

The causes of insulin resistance are not well-understood in either type 1 or type 2 diabetes. Insulin (INS) is known to undergo rapid non-enzymatic covalent conjugation to glucose or other sugars (glycation). Because the insulin receptor (IR) has INS-like regions associated with both glucose and INS binding, we hypothesize that hyperglycemic conditions may rapidly glycate the IR, chronically interfering with INS binding. IR peptides were synthesized spanning IR- associated INS-binding regions. Glycation rates of peptides under hyperglycemic conditions were followed over six days using matrix assisted laser desorption/ionization-time of flight (MALDI-TOF) mass spectrometry. INS conjugated to horse-radish peroxidase was used to determine INS binding to IR peptides in glycated and non-glycated forms. Several IR peptides were glycated up to 14% within days of exposure to 20–60 mM glucose. Rates of IR-peptide glycation were comparable to those of insulin. Glycation of four IR peptides significantly inhibits INS binding to them. Glycation of intact IR also decreases INS binding by about a third, although it was not possible to confirm the glycation sites on the intact IR. Glycation of the IR may therefore provide a mechanism by which INS resistance develops in diabetes. Demonstration of glycation of intact IR in vivo is needed.

## 1. Introduction

We propose a novel mechanism by which insulin (INS) resistance might arise in both type 1 (T1DM) and type 2 (T2DM) diabetes: Rapid glycation of the insulin receptor (IR) may result in decreased INS binding and subsequent impairment of IR activity. We test the plausibility of this hypothesis in vitro using mass spectrometry to follow the glycation of IR-derived peptides, an enzyme-linked-insulin binding assay to measure INS binding to these peptides, and ultraviolet spectroscopy to measure binding of INS to intact, glycated IR.

While development of insulin resistance is an important concomitant of the development and progression of both T1DM and T2DM, its causes are still mysterious. Insulin resistance is characterized by the presence of normal numbers of insulin receptors (IR), significantly increased levels of insulin (INS), but decreased INS activity. Various mechanisms have been proposed to explain insulin resistance. In T1DM, insulin resistance is thought to be due to hyperglycemia producing down-regulation of glucose transporters and the disruption of fatty acid regulation producing post-transport regulation of insulin activity [1,2]. Since a third of type 1 diabetics share risk factors for type 2 diabetes, such as obesity, it is possible that insulin resistance in T1DM has additional causes shared with T2DM [3]. The possible causes of insulin resistance in T2DM have been attributed variously to neural and endocrine mechanisms, inflammatory mechanisms, and cell-intrinsic mechanisms, all of which are physiologically interconnected. As in T1DM, increased serum concentrations of fatty acids (FAs), and their subsequent metabolites may trigger inhibitory phosphorylation of insulin signaling pathway elements [4]. Adipokines, including leptin, adiponectin, and resistin, are also believed to be involved [5] through changes in whole body metabolism, inflammation, drive to exercise, and hunger [6,7]. Inflammatory molecules, such as tumor necrosis factor (TNF) α and IL-6 may contribute to insulin resistance by activating various kinases that initiate phosphorylation of IR substrates [8,9,10]. Cell-intrinsic mechanisms encompass development of ectopic fat [11], which may produce an autocrine version of the aforementioned FA-based endocrine signaling changes. Other intrinsic mechanisms include oxidative stress and dysfunction of organelles, such as the mitochondria and the endoplasmic reticulum [12,13]. Thus far, none of these mechanisms have proven sufficient to explain insulin resistance. We suggest that non-enzymatic glycation of the IR may explain aspects of insulin resistance that have remained unexplained.

While non-enzymatic glycation of some proteins such as insulin (INS) and hemoglobin A1C is a well-known phenomenon accompanying hyperglycemia in both type 1 and type 2 diabetes [14,15], the effects of such glycation on receptor proteins such as the IR have not yet been explored. In fact, the extent of glycation in normal individuals is wide, encompassing several thousand unique proteins, although only a small number of these differentiate diabetic from healthy individuals [16,17]. The mechanistic effects of protein glycation on diabetes-related functions have been investigated for only a handful of these differentiating proteins: serum albumin [14,18]; CD59, an inhibitor of the membrane attack complex (MAC), which targets cells for destruction and is increased in various tissues of diabetic patients [19]; low density lipoproteins [20]; lens proteins such as α crystallins [21]; insulin-like growth factor binding protein 3 [22]; β-2 microglobulin [23]; sorbitol dehydrogenase [24]; collagen [25]; glutathione reductase [26]; and insulin (reviewed below). No one has yet investigated whether the IR or other receptors become glycated, nor whether such glycation might inhibit glucose regulation.

We hypothesize that glycation of the IR provides a logical mechanism for producing INS resistance. Our hypothesis is an extrapolation from the work of several groups that have previously demonstrated that INS itself undergoes rapid glycation under hyperglycemic conditions combined with the facts that the IR shares sequence homologies with INS and that the IR itself binds glucose.

INS glycation is well-characterized. Glycation is defined as the non-enzymatic covalent conjugation of glucose (or other sugars) to a peptide or protein. In solution, hexoses exist in equilibrium as two primary isomeric forms: the open-chain aldohexose and cyclic hemiacetal. In the presence of dissolved peptide, the aldohexose is usually susceptible to nucleophilic attack by terminal α- or lysine residue ε- amino groups. This reaction results in condensation of an unstable glycosylamine adduct, which rearranges over time into the Amadori aminodeoxyketose. The Amadori product becomes the basis for the development of advanced glycation end products (AGEs) that are highly associated with diabetic complications [27]. It is generally assumed that AGEs develop over weeks or months. The glycation of INS is extraordinary, although not unique, in taking place over hours rather than weeks [28]. A similar effect is observed in hemoglobin A1D (not to be confused with A1C), a hemoglobin fraction that glycates extremely rapidly in response to acute changes in plasma glucose [14].

Evidence suggests that the initial glycation of INS by glucose occurs at the phenylalanine 1 at the N-terminus of the B chain of INS [28,29,30,31]. With longer incubations, an average of two glycations occurs per INS at either of two additional glycation sites: the N-terminus glycine 1 of the A chain or the single lysine 29 of the B chain [30,31]. The rapidity of the glycation of insulin and the preference for glycation of particular sites is consistent with other research that has demonstrated the existence of six glucose binding sites on INS [32,33,34], including one involving the phenylalanine 1, valine 2 and glutamine 4 at the N-terminus of the B chain [34,35,36], another involving the lysine 29 of the B chain [34], and a third at the glycine 1 and glutamine 5 of the A chain [34,35]. The three other binding sites are scattered across the A and B chains [34] and involve one or more tyrosine residues, including the tyrosine 14 of the A chain [36] and histidines. Cheng and Kawakishi [37] have demonstrated that the histidines on insulin can be glycated in the presence of copper ions. d-galactose, d-mannose and 2-deoxy-d-glucose bind with lower affinity to these glucose binding sites [36]. INS can also be glycated by fructose [38].

**Figure 1:**
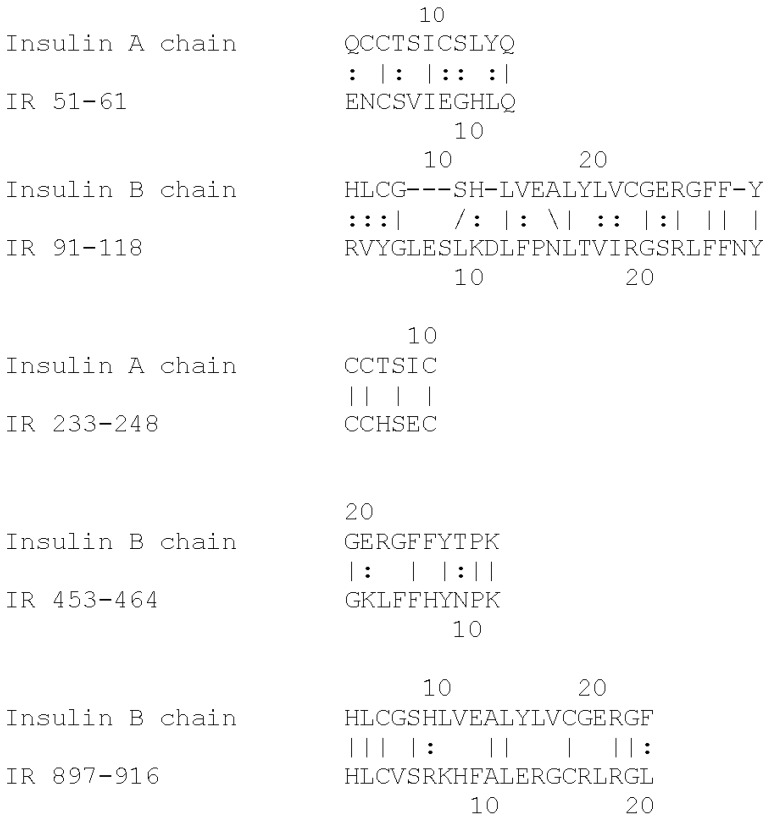
Insulin homologies in the human insulin receptor (IR). Lines indicate identical amino acids; dots indicate conserved amino acid substitutions.

**Figure 2:**
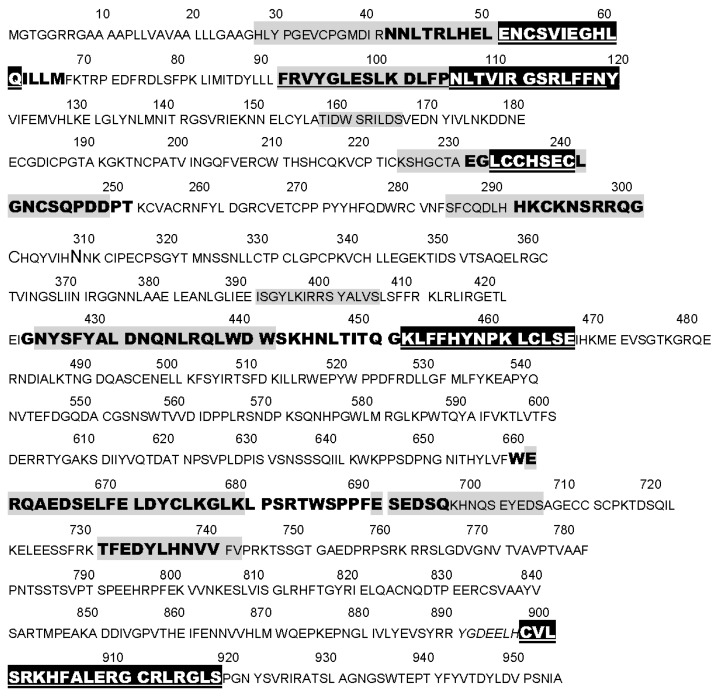
Annotated sequence of the extracellular (amino acids 1–956) portion of the insulin receptor (IR) (UniProt P06213 at www.expasy.org) summarizing where various types of studies have identified insulin binding regions. Bold letters represent sequences for which several techniques (alanine substitutions, blocking antibodies, photo-crosslinking studies—reviewed in [42]) have demonstrated insulin binding. Regions highlighted in black with white lettering mimic insulin and bind glucose (reviewed in [42]). Regions highlighted in grey mimic glucagon. Underlined regions are those that bind insulin-HRP in the present study.

Based on the extensive evidence that INS can be glycated rapidly, and the fact that the IR shares a number of INS-like regions [39,40], we hypothesize that the IR may also glycate rapidly. The regions shared by the IR with INS are summarized in Figure 1 and Figure 2. These INS-like IR regions are associated with INS binding sites (Figure 2) and also bind glucose, modifying the affinity of INS for the IR [41,42]. Moreover, INS binding to the IR is sensitive to acute variations in glucose concentration so that under hyperglycemic conditions, the binding of INS to the IR is decreased significantly [41,42]. These observations suggest that hyperglycemia could lead to glycation of INS-binding INS-like regions of the IR resulting in a detrimental effect on INS binding.

If INS and the IR both glycated rapidly under hyperglycemic conditions, then a combination of glycated INS and glycated IR could produce significantly reduced IR activation even in the presence of normal amounts of IR and stimulate the over-expression of INS that is observed in diabetes. This prediction extrapolates from the observation that INS glycation by itself results in decreased INS activity in several in vitro and in vivo mouse studies [43,44,45] and is present in poorly controlled type 1 and type 2 diabetic patients [46]. Similar rapid glycation of IR would, we predict, further inhibit INS activity, but have more pronounced and longer-acting effects than glycated INS since IR turnover is at a much slower rate of weeks [47]. Additionally, like INS, IR is stored intracellularly prior to use, and it is also recycled, so that glycation under hyperglycemic conditions might occur not only due to exposure to extracellular glucose but also intracellularly due to exposure to polyols such as sorbitol or fructose [48].

The purpose of the research presented here is to test the plausibility of this IR-glycation hypothesis by examining whether peptides derived from the IR glycate in vitro, comparing their rates of glycation to that of INS, and determining whether such glycation alters INS binding to these IR peptides and to intact IR.

## 2. Results

In order to test our hypothesis that the IR may rapidly glycate under hyperglycemic conditions, peptides were synthesized covering about half of the IR sequence (Table 1). Some of these peptides mimic INS, which is known to glycate rapidly under hyperglycemic conditions, while some of the peptides were from regions of the IR that showed no similarity to INS. The glycosylamine and Amadori adducts that result from rapid glycation have a theoretical weight of 162 da, so glycated peptide can easily be demonstrated by mass spectrometry, hence matrix assisted laser desorption/ionization-time of flight (MALDI-TOF) mass spectrometry was used to measure peptide glycation.

**Figure 3:**
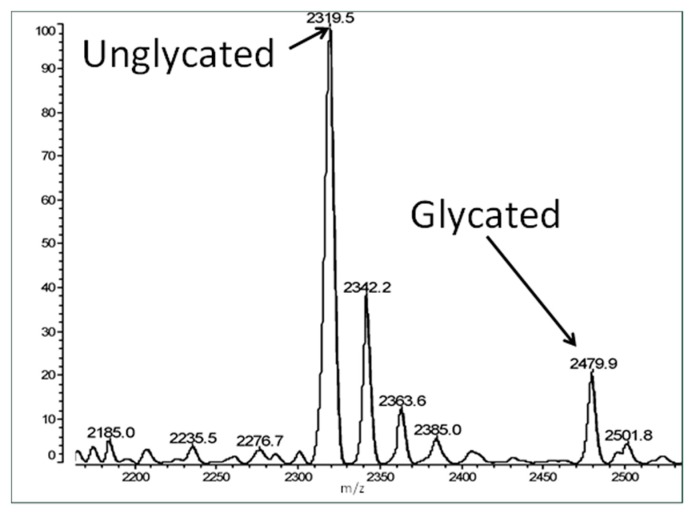
Matrix assisted laser desorption/ionization-time of flight (MALDI-TOF) spectrum illustrating non-enzymatic glycation of insulin receptor peptide 897–916 glycated in 20 mM glucose for 6 days. The unglycated peptide peak is at 2319.5 amu. Peptides were desalted with 0.1% TFA prior to analysis but some salt remained. Thus multiple peaks are observed immediately adjacent to the most prominent peak at intervals of 11 amu, which represent ionized sodium adducts produced by the desorption/ionization event in MALDI. An additional peak is observed at 160 amu (the mass of the glucose adduct) from the unglycated peptide peak, which represents the glycated peptide.

**Figure 4:**
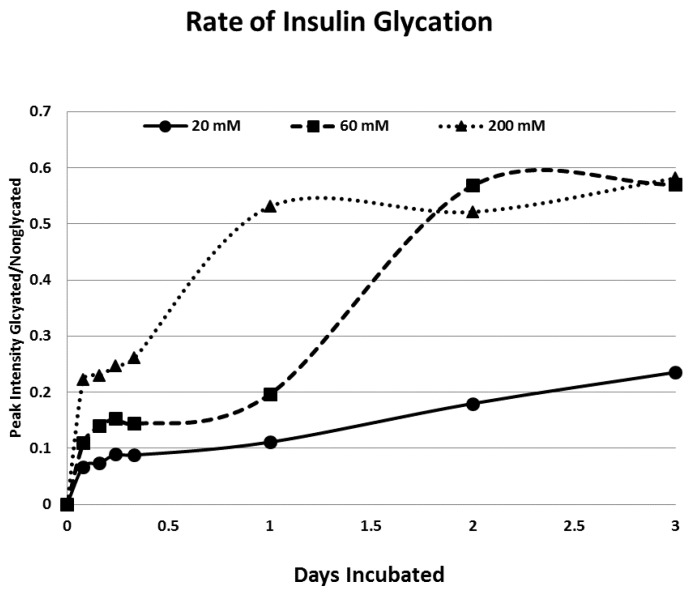
Rate of insulin glycation at various concentrations of glucose as determined by MALDI-TOF. Note that the glycation is a two-step process with a very rapid glycation occurring during the first hours of exposure to glucose and a slower glycation process occurring over several days. Data is plotted as a running average of multiple measurements at each time point.

**Figure 5:**
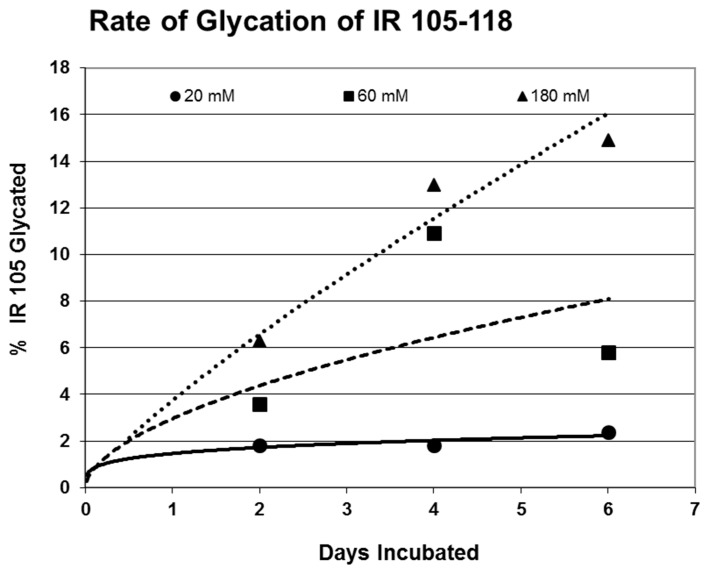
Rate of glycation of IR 105–118 at various concentrations of glucose as determined by MALDI-TOF.

**Figure 6:**
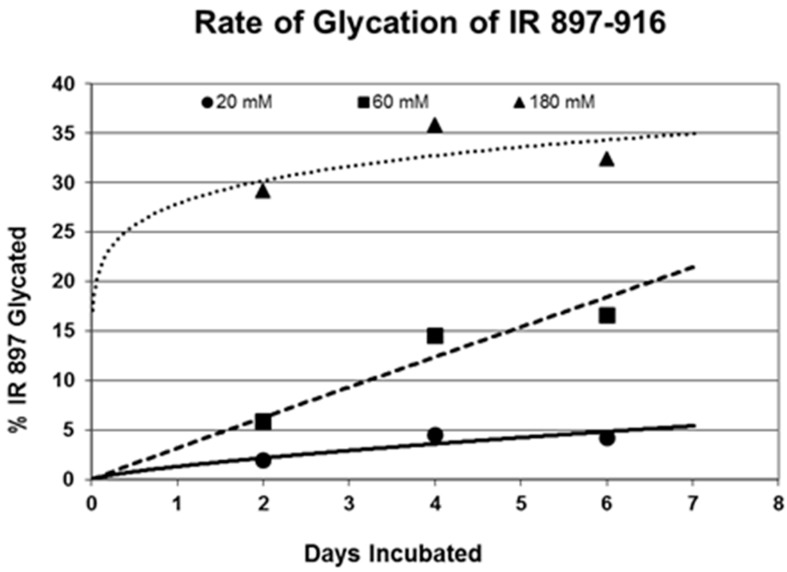
Rate of glycation of IR 897–916 at various concentrations of glucose as determined by MALDI-TOF.

Figure 3 shows an example spectrum of glycated recombinant human insulin obtained by MALDI-TOF MS. The percent glycation of total available peptide after six days exposure to 20 mM glucose is shown in Table 1 for insulin and the fifteen IR peptides tested. Ten of the peptides glycated between 2.0% and 13.5% after five or six days of exposure to 20 mM glucose. Examples of the mass spectrometry data are provided in Figure 4, Figure 5 and Figure 6 for insulin, IR 105–118, and IR 897–916.

Rates of glycation for the various IR peptides were calculated at varying glucose concentrations, as shown in Figure 4, Figure 5 and Figure 6. In no case did 5 mM glucose produce observable glycation. Glycation of IR peptides required hyperglycemic conditions. The first stage of glycation—formation of the glycosylamine adduct—is fully reversible, and free sugar and peptide likely predominate at equilibrium, since the non-reacting cyclic hemiacetal is heavily favored over the aldohexose in solution (99:1). Meanwhile, the formation of the Amadori product is less reversible, siphoning away the glucosamine adduct by rearrangement to drive the reaction forward. However, we observed an apparent asymptote in the extent of glycation at all concentrations of glucose. This is unlikely to be the result of depletion of available glucose, since concentration of dissolved glucose greatly exceeded peptide. The slow Amadori rearrangement is most probably the limiting factor. If this is the case, a majority of peptide-reacted glucose is still in the glucosamine form at six days, raising the possibility that the process is reversible with appropriate intervention (see Discussion). Glycation was not, however, complete within the six days over which these experiments were run, suggesting that continued exposure to hyperglycemic conditions can continue to glycate additional IR sites over an extended period of time measured in weeks or months if euglycemia is not established.

Glycation reactions often occur on free amines (see Introduction), but five of the fifteen peptides did not glycate measurably, although all fifteen had a free N-terminus. Similarly, glycation did not correlate with whether the peptide had a lysine residue, which also contains a free amine (Table 1). Thus, glycation is not a function merely of the presence of a free amine, but must be regulated by other aspects of the specific sequence of the peptide. For those peptides that were glycated, glycation increased in a concentration-dependent fashion from 20 mM through 180–200 mM glucose.

One factor determining the susceptibility of an IR peptide to glycation was whether it binds glucose. There was good correlation between glucose binding affinity (as summarized in Table 1 and reported previously in [41,42]), whether a peptide glycated, and the extent to which it glycated (Table 1). A few IR peptides become doubly glycated (Table 1). At the highest glucose concentrations, INS becomes tri-glycated. The percent of glycated INS we observed, and the rate at which glycation proceeded (Figure 4), compares favorably with those reported previously from experiments on INS run under similar conditions [28,29,30,31]. The rate of glycation of IR peptides under the same conditions is only slightly slower than the glycation of INS (compare Figure 4, Figure 5 and Figure 6) and hundreds of times faster than that reported for hemoglobin A1C [14].

**Figure 7:**
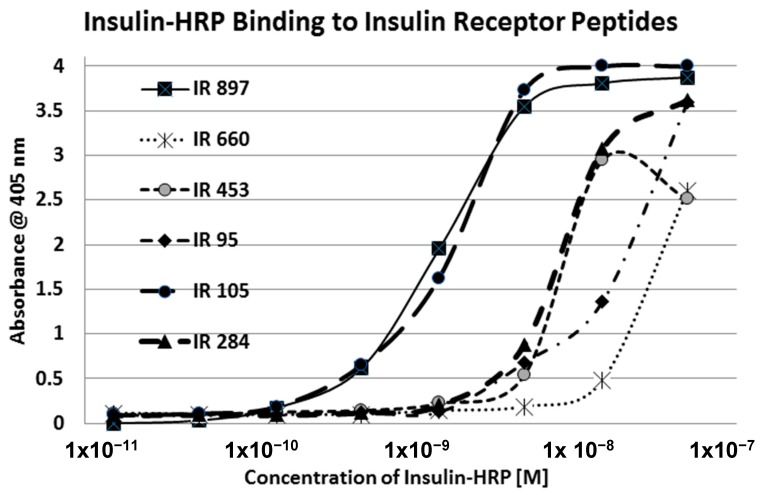
Binding curves for insulin–HRP binding to some of the insulin receptor (IR) peptides utilized in these experiments. Sequence numbers of the IR refer to the Table 1, which contains additional data. See also [41,42].

**Figure 8:**
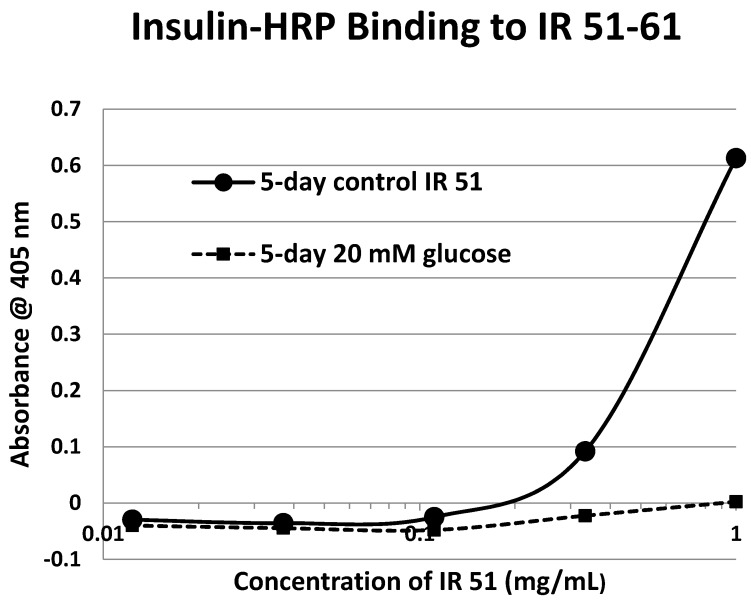
Insulin–HRP binding to insulin receptor peptide (IR) 51–61 in the absence and presence of glucose over six days. Moderate glycation in 20 and 60 mM glucose reduces the binding constant by a factor of approximately 2. Extreme levels of glycated peptide resulting from incubation in medium containing 200 mM glucose reduce binding capacity to nearly undetectable levels.

**Figure 9:**
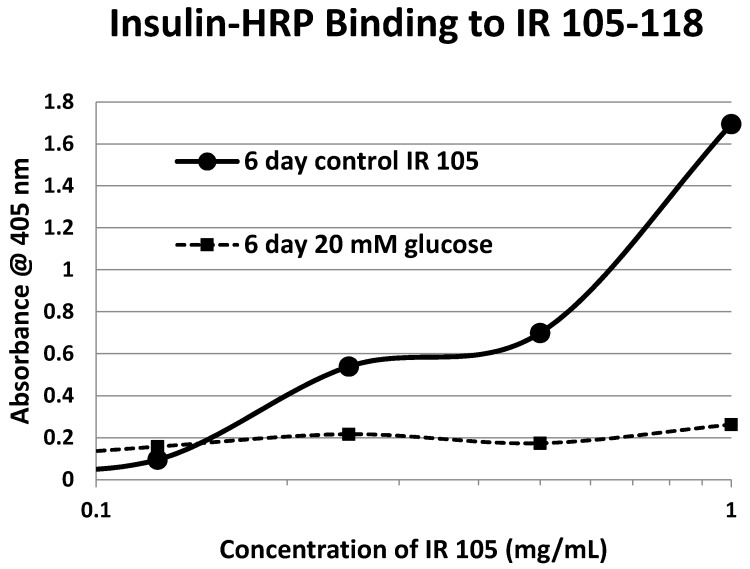
Insulin–HRP binding to insulin receptor (IR) 105–118 in the absence and presence of glucose over six days.

**Figure 10:**
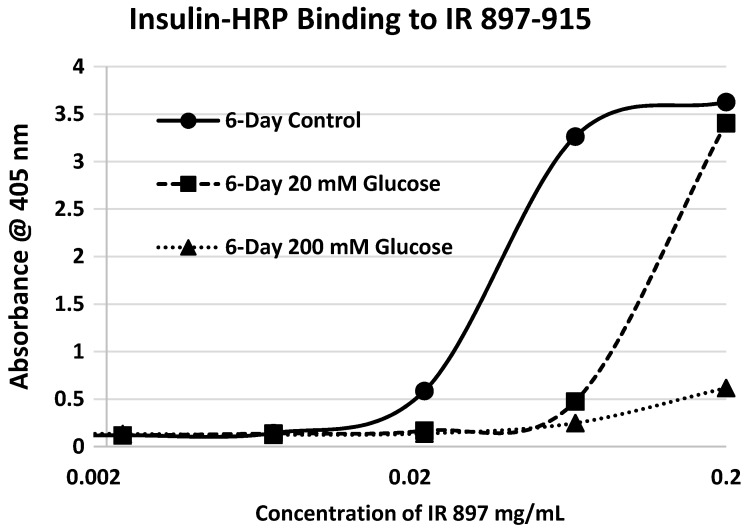
Insulin–HRP binding to insulin receptor (IR) 897–915 in the absence and presence of glucose over six days.

Our hypothesis predicts that IR glycation should interfere with INS binding to the IR. As a first test of this prediction, we examined horse-radish peroxidase-linked INS binding to IR peptides. INS-HRP binding curves for representative IR peptides are shown in Figure 7 and all binding constants provided in Table 1. Of the ten peptides that glycated measurably, only four also bound INS-HRP (IR 51–61; IR 91–103; IR 105–118; IR 897–916) (Table 1). Each of these four peptides showed very significant loss of INS-HRP binding after glycation with shifts of the binding curves to the right (Figure 8, Figure 9 and Figure 10). It is likely that steric interference of peptide-insulin bonding by the hexose-derived adduct is the source of affinity loss.

**Figure 11:**
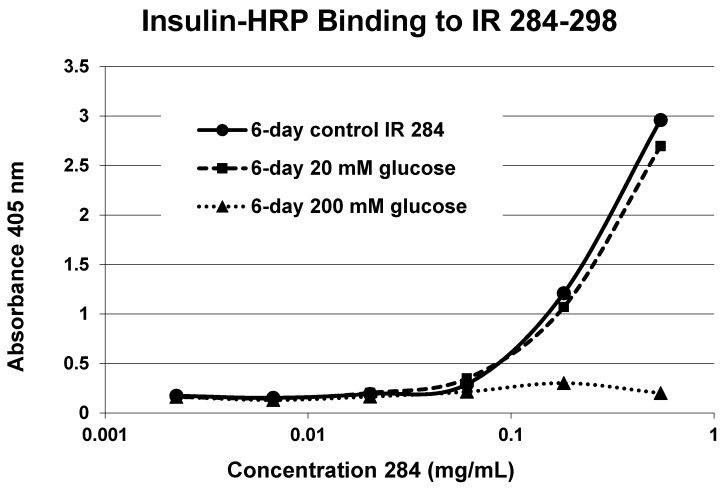
Insulin-HRP binding to insulin receptor (IR) 284–298 in the absence and presence of glucose over six days. 20 mM glucose incubation had a negligible effect on insulin-HRP binding, while 200 mM glucose shifted the curve dramatically to the right.

A positive control confirms that the affinity loss is likely due to peptide glycation and not other possible factors. INS-HRP bound to one of peptides (IR 284–298) (Figure 11) that did not glycate. The same 6-day exposure to 20 mM glucose that significantly altered INS-HRP binding to the four other IR peptides did not alter the affinity of IR 284–298 for INS–HRP. Thus, glycation, rather than peptide degradation or other possible factors, is almost certainly the cause of the loss of INS binding to the glycated peptides.

**Figure 12:**
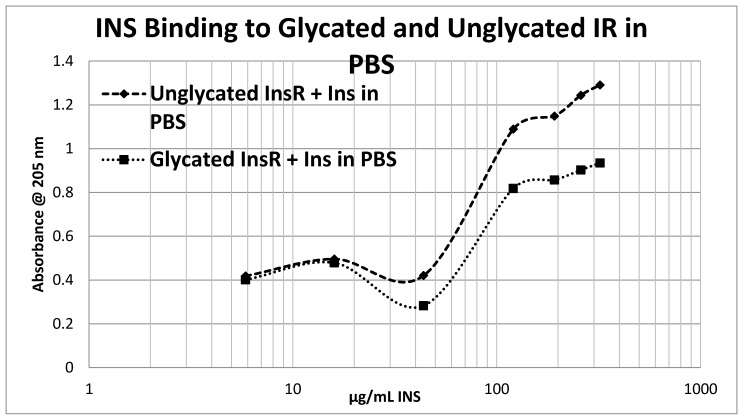
Insulin (INS) binding to glycated and unglycated insulin receptor (InsR) in phosphate buffered saline without glucose. The binding constant of the INS for InsR under these conditions was found to be 70 μg/mL of INS. Glycation reduced the amount of binding by almost exactly one third without appreciably altering affinity.

**Figure 13:**
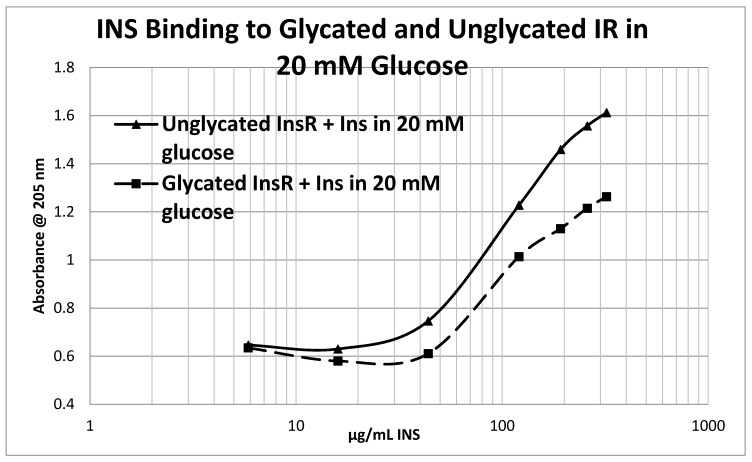
Insulin (INS) binding to glycated and unglycated insulin receptor (InsR) in phosphate buffered saline with 20 mM glucose. The binding constant of the INS for InsR under these conditions was found to be 110 μg/mL of INS (compared with 70 in the absence of glucose; see Figure 12). Glycation reduced the amount of binding by almost exactly one third without appreciably altering affinity.

**Figure 14:**
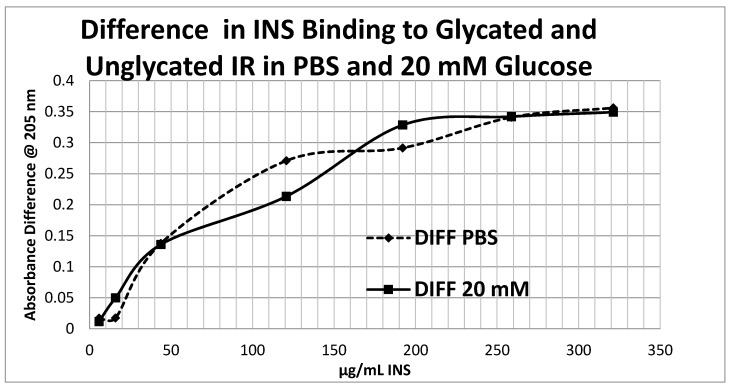
The difference in insulin (INS) binding to glycated and unglycated insulin receptor (IR) in PBS with and without 20 mM glucose. Note that although the binding constants of INS binding under these conditions differed (see Figure 12 and Figure 13), the effect of glycation on decreasing INS binding was the same. Thus, the diminution in INS binding to glycated IR is independent of the presence or absence of free glucose.

Glycation of the intact insulin receptor (IR) also resulted in decreased INS binding to the receptor as demonstrated in Figure 12, Figure 13 and Figure 14. In this case, we employed ultraviolet spectroscopy to measure INS binding to the IR, using concentration-dependent shifts in the spectra to calculate binding curves. INS and IR were separately incubated in either phosphate buffered saline at pH 7.4 (PBS) or 20 mM glucose in PBS for six days and then the binding of unglycated and glycated INS was determined with both unglycated and glycated IR. The binding constant of unglycated INS for unglycated IR in PBS was determined to be 70 µg/mL of INS (Figure 12), whereas the binding constant of unglycated INS for unglycated IR in 20 mM glucose was determined to be 110 µg/mL of INS (Figure 13), confirming our previous report that hyperglycemic conditions decrease INS binding to the IR [40,41]. Glycation of the IR decreased INS affinity for the IR in both PBS and 20 mM glucose (Figure 12 and Figure 13), shifting the curve to the right. More significantly, glycation decreased the *amount* of binding by almost exactly one third (as measured by the decrease in absorbance) in both PBS and 20 mM glucose (Figure 12 and Figure 13). Figure 14 shows that the amount of binding decrease due to IR glycation is the same whether the medium contained glucose or not.

Overall, these experiments demonstrate that INS binding to the IR exposed to hyperglycemic conditions results in significant decreases in both affinity and binding of INS to the IR. While the presence of glucose in the medium decreases INS affinity for the IR, glycation blocks INS binding to the IR. The amount of blocking is independent of the presence or absence of glucose in the medium and must therefore be due to an irreversible alteration in IR structure (Figure 14).

One final set of tests of our hypothesis were also attempted, which involved the demonstration of glycation on intact IR isolated from rat liver and exposed to hyperglycemic conditions. Attempts to identify the sites of glycation on intact IR using proteolytic digestion followed by mass spectrometry (MS) have so far been unsuccessful. Those regions of the IR containing peptides most likely to be glycated according to the data summarized in Table 1 are consistently missing from the MS data output, although all of the rest of the protein is properly reported. The missing data may indicate that these regions are not properly proteolytically digested due to protection of digest sites by glycation; that glycation neutralizes the fragments so that they remain uncharged and are therefore not analyzed; that the resulting fragments are altered sufficiently to go unrecognized by the peptide identification program; or may be due to some other unknown cause. We continue to attempt to resolve this hiatus and note that this problem may explain why IR glycation has not been reported previously by other MS-based studies of protein glycation in T1DM and T2DM.

## 3. Discussion

Our principle findings are as follows. No IR peptide glycated under euglycemic conditions of 5 mM glucose. Several IR peptides were glycated up to 10% within hours of exposure to 20–60 mM glucose but did not reach maximal glycation even after six days of glucose exposure. The rate of glycation is far more rapid than that observed with hemoglobin A1C and directly comparable to that of INS and hemoglobin A1D [14]. Glycation was not associated with free terminal amines or lysine residues, but correlated with the presence of previously demonstrated glucose binding sites on the IR peptides. Thus, glycation of the IR is dependent on local amino acid sequences that determine the glucose sensor capability of the IR [40,41]. Four glycatable IR peptide regions participate in INS binding to the IR. Glycation of these four peptides significantly inhibits INS binding to them. One peptide that also participates in INS binding, but does not bind glucose, was not significantly glycated nor was its INS-binding capacity altered under hyperglycemic conditions, thereby demonstrating that glycation is the key factor in decreased INS binding. Glycation of intact IR also results in decreased INS binding and this decrease is augmented in the presence of previously glycated INS. Thus, our hypothesis that the IR can be glycated rapidly under hyperglycemic conditions is supported as is our prediction that such glycation can interfere with INS binding to the IR. IR glycation therefore provides a plausible mechanism for producing INS resistance.

This study builds on a large body of prior literature demonstrating that INS is glycated and that such glycation leads to decreased INS function. Our data on INS glycation is completely consistent with this prior literature [38,39,40,41], helping to establish the reliability of our IR glycation data. Our study differs significantly from previous studies in examining the glycation of IR and its effects on INS binding. Our results therefore extend our current understanding of glycation reactions as a mechanism for producing INS resistance from INS to its receptor.

The strength of this study is that glycation-associated decreases in INS binding to IR peptides correlates completely with regions of the IR previously demonstrated to be involved with INS binding. Thus, there is good reason to believe that the reduction in INS binding associated with glycation will also occur in native IR in vivo. The fact that the Amadori product resulting from this glycation is irreversibly bound to the peptides may provide a mechanism to explain the observation that exposing cell cultures to hyperglycemic conditions results in an irreversible inhibition of subsequent INS activity [48]. Subsequent modification of initial glycation products into AGEs may further increase and extend the longevity of the INS resistance that results.

The main weakness of this study is that the experiments were performed under in vitro rather than in vivo conditions and that we have not demonstrated that inhibition of INS binding actually leads to decreased IR function. The use of HRP-labeled INS undoubtedly modified the affinity of the INS for IR peptides, although the result is probably to underestimate the effects of glycation on INS binding rather than the opposite. In any event, the HRP-labeled INS results were replicated using unlabelled INS on intact IR. It also remains to be investigated whether increases in concentrations of polyols such as sorbitol and fructose due to chronic hyperglycemia may produce intracellular glycation of IR. Unfortunately, as noted in the Results section, our attempts to measure glycation of intact IR using mass spectrometry techniques have thus far failed due to the disappearance of a significant fraction of the IR during analysis. Notably, the specific fraction that goes missing consists exactly of those portions of the IR that our peptide studies demonstrate to be glycated. Until we resolve this analytical issue, tissue or animal studies exploring the possible effects of glycation in vivo will not be possible.

**Figure 15:**
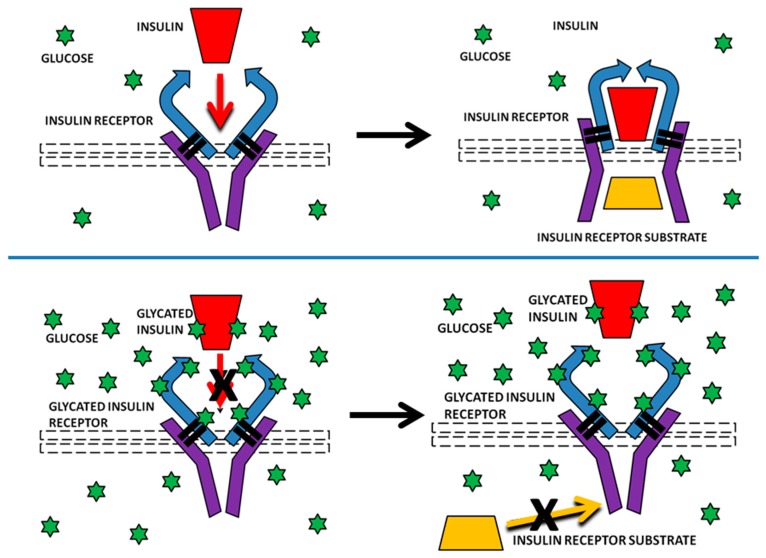
A summary of the mechanism proposed here by which glycation of insulin (INS) and the insulin receptor (IR) results in decreased insulin activity. TOP: At normal concentrations of glucose (green stars), INS (red block) binds to the IR (extracellular chains in blue and intracellular chains in purple lines) causing conformational changes that result in the binding of insulin receptor substrate (IRS) (yellow block) and second messenger activation. BOTTOM: Under hyperglycemic conditions, INS and the IR both glycate. Glycated INS has reduced affinity for the IR and reduced activity and glycated IR has reduced affinity for INS. A combination of glycated INS and glycated IR can be expected to produce greatly reduced binding to the IR and, therefore, significantly decreased IRS binding and second messenger activation. This reduced activation will be short-lived for INS, which turns over rapidly (on the order of every 30 min) but will produce chronic insulin resistance through glycated IR, which turns over only every month or so.

Our results are potentially important for providing a possible mechanism by which INS resistance can be caused directly by hyperglycemia, without obviating or disproving other pathways that may also contribute to insulin resistance, such as those reviewed in the Introduction. Specifically, glycation of INS itself has already been demonstrated to reduce its affinity for the IR and IR activation and we have demonstrated here that IR glycation decreases INS binding. In combination, glycation of both INS and IR would result in a significant decrease in INS binding to the IR (one third or more according to our data), thereby inhibiting IR-mediated INS signaling. Because the IR turns over on the order of every several weeks or months, the effects of IR glycation from even transient hyperglycemia would be expected to be chronic in nature. Repeated hyperglycemic episodes, or chronic hyperglycemia could result in severe and very long-term inhibition of IR activity through such glycation (Figure 15).

Additionally, we emphasize that other proteins share sequence homologies with INS and IR, including insulin-like growth factors (IGF), IGF-binding proteins [22], IGF receptors (IGFR), glucagon receptors, and glucose transporters (GLUT) [41,49]. Any or all of these proteins may also glycate, further disrupting glucose homeostasis.

## 4. Materials and Methods

### 4.1. Insulin Receptor Peptides

Insulin receptor peptides (Figure 1) were synthesized by RS Synthesis (Louisville, KY, USA) to at least 95% purity by high pressure liquid chromatography (HPLC) and mass spectrometry. Peptides or recombinant human insulin (rHu) (Lilly, Indianapolis, IN, USA) were dissolved in glucose-free, phenol-red-free, Dulbecco’s modified Eagle growth medium (DMEM) to 1 mg/mL. Stock 2.0 M d-glucose (Sigma-Aldrich, St. Louis, MO, USA) solutions were also made in DMEM. The peptide and glucose stocks were combined to yield final reaction solutions with peptides at 0.1 mg/mL and glucose at 0.0, 5.0, 20, 60, 200 or 2000 mM. Combinations were incubated at 36–38 °C for 1–6 days. Reactions were stopped and solutions were stored by freezing at −20 °C. Glycated peptides were desalted by centrifugal filtration through Ultrafree-MC 5 kda or Ultracel 3 kda filter units. Resulting retentate was resuspended in low ionic strength saline immediately before performing mass spectrometry.

### 4.2. Mass Spectrometry

Samples were analyzed by matrix-assisted laser desorption/ionization time-of-flight mass spectrometry on a Shimadzu Axima MALDI-TOF MS. The 256-well MALDI plate was prepared by washing with 3:1 methanol/acetonitrile (ACN). In each well, the peptide layer was sandwiched between layers of matrix (saturated α-cyano-4-hydroxycinnamic acid in 3:1 ACN/0.1% TFA) to maximize ionization. The peptide layer was desalted on plate with three aliquots of 0.1% trifluroacetic acid (TFA) prior to adding the final layer of matrix. 300 laser firing/detection events were averaged per final spectrum, and low molecular weight compounds were excluded by ion gating to avoid overwhelming the mass analyzer with small molecules. The extent of glycation was calculated as a percent of the average of three experiments, by signal height, of the unglycated peptide peak. The percent was graphed against incubation time, and fit to a power function.

### 4.3. Insulin-HRP Binding to IR Peptides

Each peptide was held constant at 0.1 mg/mL in pH 7.4 phosphate-buffered saline, and 100 μL aliquots were pipetted into rows of a Costar high-binding ELISA plate and incubated for an hour. The plate was washed 3× with 0.1% Tween 20 (Sigma-Aldrich) in 0.05 M phosphate buffered saline (PBS). Two hundred µL 2% polyvinyl alcohol in PBS were added to each well and incubated 1 h. The plates were then washed 3× again with 0.1% Tween 20. A solution of insulin conjugated to horse-radish peroxidase (HRP), 0.83 µg in 1.0 mL PBS, was made and serially diluted with PBS by thirds, ten times. These serial dilutions were added to the plate wells, incubated for an hour, and then washed 3× as before. Finally, 100 µL of stock ABTS [2,2′-azino-bis(3-ethylbenzothiazoline-6-sulphonic acid)] (Millipore, Burlington, MA, USA) was added to each well, incubated for thirty minutes, and the plate was then read on a SpectraMax Plus scanning microplate spectrophotometer at 405 nm. Controls wells lacking peptide but with PVA, INS-HRP and ABTS measured non-specific binding that was subtracted from the absorbance values of the rest of the wells. All experiments were run in triplicate and the averages of the corrected absorbances were then plotted against the concentration of the INS–HRP for each peptide to yield binding curves. Because binding curves are calculated by plotting differences, it was not possible to incorporate error bars into these curves.

### 4.4. Effect of Glycation of Insulin-HRP Binding to Peptides

The same technique just described in Section 4.3 was used to measure the effect of glycation on INS-HRP binding to IR peptides as above with one change: IR peptides were serially diluted instead of the INS-HRP, which was held constant. This methodological modification was made in order to reduce the amount of IR peptide required to obtain results. As in Section 4.3, each experiment was run in triplicate and the results averaged.

### 4.5. Effect of Glycation of Insulin Receptor on Insulin Binding

Intact recombinant IR was obtained from Sigma-Aldrich (St. Louis, MO, USA) and utilized at 42.9 units/mL in phosphate buffered saline, pH 7.4 (PBS) or in PBS with 20 mM glucose added. PBS and 20 mM glucose solutions of IR were incubated for six days at 37 °C. An aliquot of each preparation was then subjected to centrifugal filtration and washing through Ultrafree-MC 5 kda or Ultracel 3 kda filter units. The resulting retentates were resuspended in PBS. In this way, identical preparations of unglycated IR and glycated IR were made in both PBS and in 20 mM gucose PBS. Microliter quantities of PBS buffer, or 20 mM glucose PBS, or human recombinant INS (Sigma-Aldrich, St. Louis, MO, USA) in PBS or 20 mM glucose PBS were then added serially to 100 µL of each of the four preparations of IR and to control wells containing only PBS or 20 mM glucose PBS. Experiments were carried out in a crystal 96-well plate and the serial combinations subjected to UV spectrophotometry to obtain their spectra. The spectra of the control wells were subtracted from the spectra of the experimental wells to yield expected values at each UV wavelength and differences between the controls and experimental values determined. These differences were plotted according to the concentration of INS added to the IR to yield binding curves. Binding constants were determined from the concentration at which the binding curves inverted. Because of the small amount of retentates, the various conditions were not run in duplicate; instead, the experiment was run independently, twice. Each run yielded similar binding constants, but because the data from each run had different baseline values, it was not possible to average the results, so only one of the sets of results is presented here.

## 5. Conclusions

The IR rapidly glycates under hyperglycemic conditions resulting in significantly decreased INS binding under in vitro conditions. If this effect also occurs in vivo, the clinical implications of rapid glycation under hyperglycemic conditions may be to cause interference with binding of INS, IGF, and related hormones to their receptors that would last for the lifetime of the ligands and their receptors. Direct measurement of IR, IGFR, and glucagon receptor-glycation might provide more accurate and sensitive means for evaluating the effects of hyperglycemic episodes in diabetes than does hemoglobin A1C.

Understanding glycation mechanisms of INS-related proteins such as the IR and IGFR may provide new means for pharmacologically or nutritionally mitigating some adverse effects of hyperglycemic episodes. Since glycation of IR peptides is not complete at six days under hyperglycemic conditions, and the initial steps are reversible, various interventions may be possible to block glycation. Various investigators have reported that vitamin C, aspirin, lysine, and other natural compounds can inhibit INS or human serum albumin glycation in vitro or in vivo [50,51,52]. In addition, dehydroascorbic acid (the oxidation product of vitamin C) has been demonstrated to compete with glucose uptake through glucose transporters, thereby lowering decreasing the intracellular effects of hyperglycemia on sorbitol production [48,53]. If these results are robust, then we would expect these and related anti-glycosylation agents [54,55] to be effective for decreasing insulin resistance by decreasing glycation of IR, IGFR, INS, IGF, and related proteins.

In summary, IR glycation due to hyperglycemia may broaden the significance of protein glycation for understanding insulin resistance well beyond the existing literature on INS, hemoglobin, and serum albumin by providing a direct mechanism for lasting effects of acute hyperglycemic episodes on IR function. These results may extend to other INS-related proteins such as IGF and the IGFR. Better understanding of rapid non-enzymatic glycation of such proteins may provide better clinical diagnostic measures as well as lead to specific treatment interventions.

## Figures and Tables

**Table 1:** Table summarizing experiments to identify motifs determining insulin-receptor (IR) peptide glycation and whether glycation of IR peptides results in inhibition of insulin binding to these peptides. Specifically, this table provides binding constants glucose binding non-covalently to various insulin receptor (IR) peptides; glycation of the same IR peptides at 20 mM glucose for six days; the binding constants (µM) of insulin-HRP (Ins-HRP) to IR peptides from (40, 41) binding of Ins-HRP at 10 nM to the IR peptides in the absence of glycation; and whether there is a change in Ins-HRP binding at 10 nM to each IR peptide after exposure to glycating conditions (six days at 20 mM glucose). Some peptides glycated at more than one position, so the column showing “% Glycation” shows the percent of peptide glycated at the primary site followed by the percent glycated at the secondary site. The numbers of the IR peptides at the left refer to the UniProt numbering system for the human IR (P06213). The abbreviations in parentheses following the peptide number refer to whether the peptide mimics insulin (insulin-like or IL) or glucagon (glucagon-like or GL) (see [38,40,41]). Known enzymatic glycosylation sites (N-acetylglucosamine) are indicated by the bolded N (asparagine) with a grey background (see UniProt P06213 at www.expasy.org). Lysines (K), which are often targets of the Maillard reaction, are printed in white against a black background. Note that not all N are enzymatic glycosylation sites. Note also that there is no obvious correlation between the presence of K and glycation, nor are all peptides glycated, although free amines are present on all of the peptides and are often associated with glycation reactions (see text). Glycation, under the conditions utilized here, is better associated with the affinity of glucose for the IR peptide.

Insulin Receptor Peptides	Peptide Molecular Weight	Glucose Binding Kd (mM)	% Glycation 6 Day 20 mM	Ins-HRP Kd (µM)	10 nM Ins-HRP Binding (Native)	Ins-HRP Binding Inhibition (Glycated)
Insulin	5808	0.25 & 30.0	24/6	Not Done	Not Done	Not Done
28-43 HLYFGEVCPGMDIRNN	1906	50	0/0	ND	no	no
37-51 GMDIRNNLTRLHELE	1677	40	11/0	ND	no	no
51-61 (IL) ENCSVIEGHLQ	1316	52	6/0	ND	YES	YES
91-103 (IL) FRVYGLESLKDLF	1570	37	13/4	2.5	YES	YES
105-118 (IL) NLTVIRGSRLFFNY	1568	35	2.4/0	6.2	YES	YES
157-166 (GL) TIDWSRILDS	1202	57	4/2	>100	no	no
233-248 (IL) CCHSECLGNCSQPDD	2376	46	14/0	>100	no	no
284–300 (GL) SFCQDLHHKCKNSRRQG	1835	>500	0.1/0	3.0	YES	INSIGNIF.
390-405 (GL) EISGYLKIRRSYALVS	1510	30	13/2	55.0	no	no
425-444 (GL) YSFYALDNQNLRQLWDWSKH	2316	>500	0/0	>100	no	no
453-464 (IL) TQGKLFFHYNPK	1467	57	9/0	>100	no	no
660-679 ERQAEDSELFELDYCLKGLK	2474	70	0/0	>100	no	no
689-705 ESEDSQKHNQSEYEDS	1912	63	0/0	ND	no	no
730-742 KTFEDYLHNVVFV	1261	60	11/0	ND	no	no
897-916 (IL) HLCVSRKHFALERGCRLRGL	2314	14	4.5/0	0.3	YES	YES

## Abbreviations

INS	insulin;
IR	insulin receptor;
T1DM	type 1 diabetes mellitus;
T2DM	type 2 diabetes mellitus;
DMEM	Dulbecco’s modified Eagles growth medium
